# Donor cell‐derived chronic myelomonocytic leukemia presenting after allogeneic hematopoietic cell transplantation for T‐cell acute lymphoblastic leukemia

**DOI:** 10.1002/ccr3.3383

**Published:** 2020-10-07

**Authors:** Kevin C. Miller, Zaid H. Abdel Rahman, Ryan S. Robetorye, James M. Foran

**Affiliations:** ^1^ Department of Medicine Massachusetts General Hospital Boston MA USA; ^2^ Division of Hematology/Oncology Mayo Clinic Jacksonville FL USA; ^3^ Department of Laboratory Medicine and Pathology Mayo Clinic Phoenix AZ USA

**Keywords:** allogeneic hematopoietic cell transplant, chronic myelomonocytic leukemia, CMML, donor cell leukemia, stem cell transplant

## Abstract

Donor cell leukemia is a very rare cause of relapse after allogeneic hematopoietic cell transplantation (alloHCT). Herein, we describe an unprecedented case of donor cell‐derived chronic myelomonocytic leukemia (CMML) presenting seven years after a 51‐year‐old man received a matched‐related alloHCT from his 59‐year‐old brother for T‐cell acute lymphoblastic leukemia.

## Graphical abstract

Donor cell (DC) leukemia is a rare cause of relapse after allogeneic hematopoietic cell transplantation and usually presents as acute myeloid leukemia. Uncommonly, other DC‐derived hematologic malignancies can occur and usually have a poor prognosis.

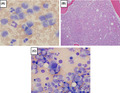

## 1 INTRODUCTION

Donor cell leukemia (DCL) is a rare cause of leukemic relapse after allogeneic hematopoietic cell transplantation (alloHCT), occurring in approximately 0.1% of transplants.^1^ DCL usually presents 1‐2 years post‐transplant with an acute myeloid leukemia (AML) phenotype, although cases have been described of donor cell‐derived myelodysplastic syndrome (MDS), acute lymphoblastic leukemia (ALL), and chronic myeloid leukemia.^2^

Postulated mechanisms of DCL include disturbed microenvironmental host factors that promote leukemogenesis, accelerated telomere shortening in hematopoietic cells after transplant, and mutated subclonal populations within the graft that predispose toward the development of further oncogenic driver mutations.^3^ These processes are similar to the biological factors that underpin de novo leukemias.

Here, we report the first case to our knowledge of donor cell‐derived chronic myelomonocytic leukemia (CMML), which arose in a patient after they received an alloHCT for T‐cell ALL (T‐ALL).

## 2 CASE REPORT

A 51‐year‐old man with no significant past medical history presented with fatigue, bilateral submandibular lymphadenopathy and was found to have acute kidney injury with a creatinine of 5.3 mg/dL. A computed tomography scan of the chest, abdomen, and pelvis showed a 6.0 × 3.5 cm mediastinal mass, small bilateral pleural effusions, and mediastinal, periaortic, and inguinal lymphadenopathy. A core needle biopsy of the mediastinal mass demonstrated blasts with immunohistochemical positivity for CD3, CD43, CD10, weak positivity for CD5, and Ki‐67 present in 95% of cells. The morphology was consistent with T‐ALL. A bone marrow aspirate and biopsy demonstrated increased cellularity with 81% lymphoblasts. Cytogenetic studies showed normal male 46,XY karyotype, and fluorescence in situ hybridization analysis for stereotypic T‐ALL genomic abnormalities was negative. The peripheral blood leukocyte count was 5.3 × 10^9^/L (with 20% lymphoblasts), hemoglobin was 12.2 g/dL, and platelets 129 × 10^9^/L. Lactate dehydrogenase (LDH) was 206 IU/L (normal range: 122‐222 IU/L).

Induction commenced with hyper‐CVAD (hyperfractionated cyclophosphamide, vincristine, doxorubicin, and dexamethasone with alternating cycles of high‐dose methotrexate and cytarabine). The cerebrospinal fluid was negative for leukemic cells. After Cycle 1A, a repeat bone marrow aspirate showed no evidence of T‐ALL. The acute kidney injury resolved with hydration and induction. After four cycles (1A‐2B) of hyper‐CVAD (4 months after the initial diagnosis), ^18^F‐fluorodeoxyglucose (FDG) positron emission tomography/computed tomography showed a reduced size of the mass to 3.3 × 2.3 cm, which was not FDG‐avid, and a repeat bone marrow biopsy was unremarkable. Induction was uncomplicated save for an acute gout flare. The patient received eight treatments with intrathecal methotrexate and cytarabine.

The patient subsequently received a matched‐related alloHCT from his 59‐year‐old brother; the donor's only known medical comorbidity was polymyalgia rheumatica. The patient's HCT Comorbidity Index score was 1; Karnofsky Performance Status was 90%. He was conditioned with a myeloablative regimen of total body irradiation (200 centiGray in six fractions), etoposide 60 mg/kg (day −3), and cyclophosphamide 100 mg/kg (day −2). He was infused with 8.0 × 10^6^ peripheral blood hematopoietic cells. Graft‐versus‐host disease (GVHD) prophylaxis was completed with tacrolimus and mycophenolate mofetil, plus one dose of anti‐thymocyte globulin (2.5 mg/kg). Engraftment of neutrophils occurred on day + 8 post‐transplant; platelet engraftment occurred on day + 14. The patient had overall grade II acute GVHD (stage 3 of skin) on day + 15, which was resolved with systemic corticosteroids. Transplant was otherwise uncomplicated save for another gout flare and a *Clostridium difficile* infection. He also developed an axonal sensorimotor polyneuropathy attributed to the conditioning chemotherapy and/or tacrolimus, after which he switched from tacrolimus to sirolimus on day + 45. Chimerism was 100% donor in CD3 and CD33 fractions on day + 30 and +60.

**Figure 1:**
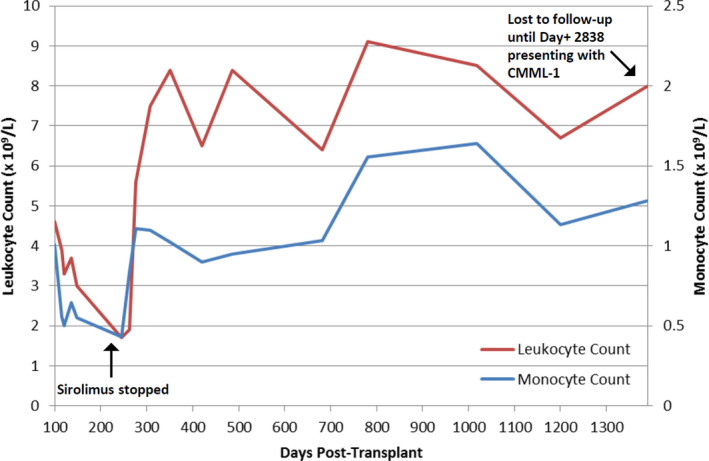
Peripheral blood counts after Day + 100 post‐alloHCT. The total peripheral blood leukocyte count is indicated in red (*y*‐axis on the left). The absolute monocyte count is indicated in blue (*y*‐axis on the right). The *x*‐axis represents days after infusion of the peripheral blood hematopoietic cell graft (ranging from Day + 100 to Day + 1388). Sirolimus was discontinued on Day + 230 given persistently low counts. The patient was lost to follow‐up after Day + 1388 for four years until presenting with overt chronic myelomonocytic leukemia on Day + 2838 (not shown). Reference range for the leukocyte count is 3.8‐10.4 × 10^9^/L. Reference range for the monocyte count is 0.2‐0.8 × 10^9^/L

A day + 100, re‐staging bone marrow showed 30% cellularity with normal trilineage hematopoiesis and 100% donor chimerism. At that time, leukocytes were 4.6 × 10^9^/L, hemoglobin 11.3 g/dL and platelets 204 × 10^9^/L. The trend of peripheral blood counts after day + 100 is shown in Figure 1. He was noted to have leukopenia over the next few months. Repeat bone marrow biopsy at day + 180 again showed 30% cellularity and no morphologic or immunophenotypic evidence of T‐ALL. Chimerism analysis showed 100% donor DNA. A complete blood count performed at that time demonstrated the following: leukocytes 1.7 × 10^9^/L, hemoglobin 10.8 g/dL, and platelets 278 × 10^9^/L; occasional pseudo‐Pelger‐Huët cells were also noted in the peripheral blood. Given unclear etiology of leukopenia, sirolimus was discontinued on day + 230, with subsequent improvement of the leukocyte count (Figure 1).

During the next three years, the patient's peripheral blood counts normalized, although his peripheral blood monocytes were persistently >1.0 × 10^9^/L (Figure 1). There was never evidence of chronic GVHD. The polyneuropathy improved with time, but he continued to require a walker to ambulate. After three years post‐transplant, the patient did not wish to follow‐up regularly at our institution.

**Figure 2:**
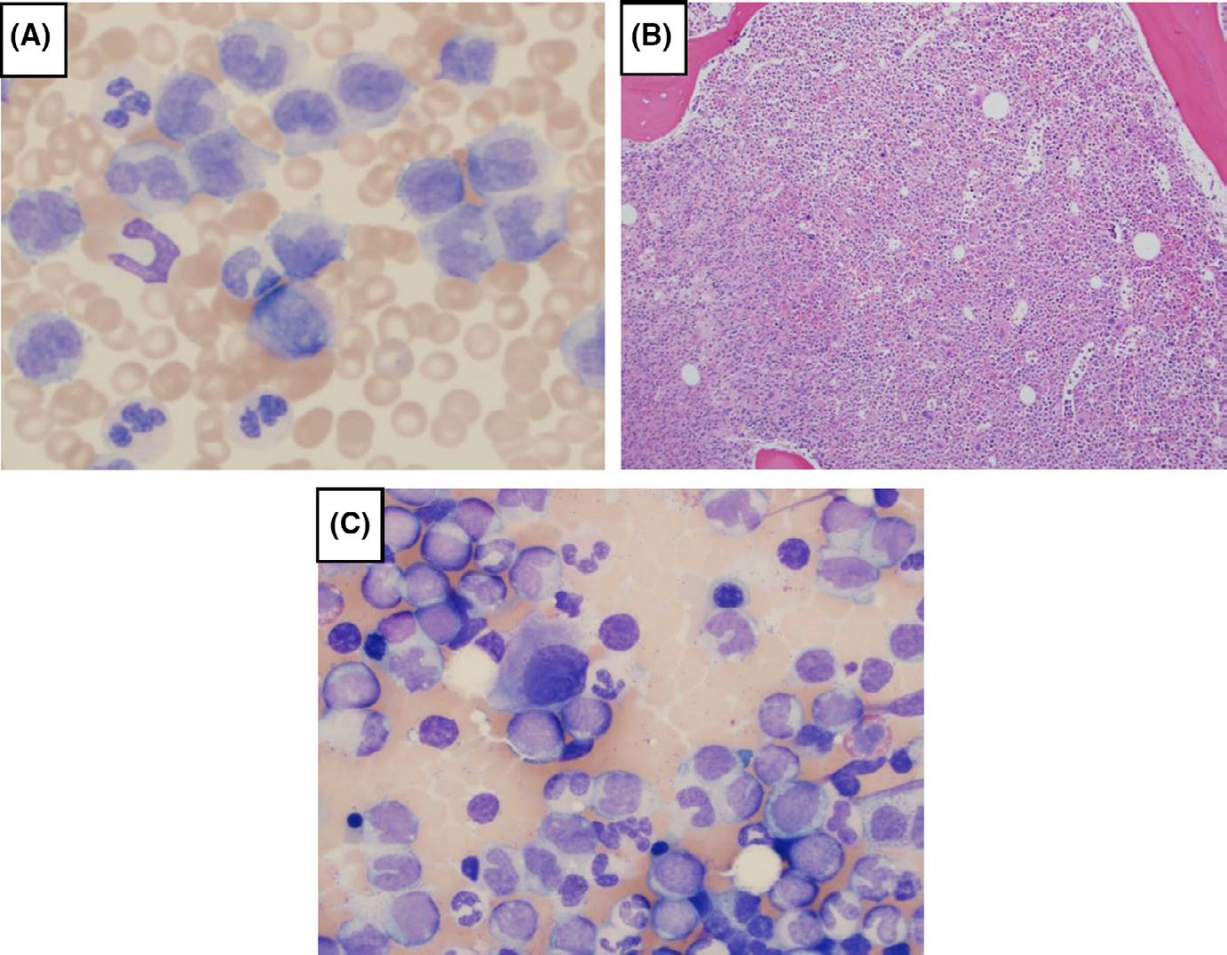
Bone marrow biopsy showed donor‐derived chronic myelomonocytic leukemia. (A) Peripheral blood monocytosis (arrows point to several monocytes; 60X magnification). (B) Hypercellular bone marrow core biopsy (greater than 95% cellular; 10X magnification). (C) Bone marrow aspirate smear with monocytosis and dyspoietic features, including monolobated megakaryocytes (arrow), and hypogranular neutrophils (arrow) (60X magnification)

Four years thereafter (7 years post‐transplant, day + 2838), the patient presented with symptoms of a urinary tract infection and was found to have a leukocyte count of 275 × 10^9^/L, hemoglobin of 12.0 g/dL, and platelets of 316 × 10^9^/L, which did not improve after antibiotic therapy. A peripheral blood smear showed absolute neutrophilia (56%) with granulocytic left shift and pseudo‐Pelger‐Huët cells, monocytosis (22%), basophilia (3%), and circulating blasts (2%) (Figure 2A). LDH was 800 IU/L, with no evidence of tumor lysis syndrome. A bone marrow biopsy showed 100% cellularity (Figure 2B), with a concomitant bone marrow aspirate exhibiting features of a myelodysplastic/myeloproliferative neoplasm, including 25% monocytes and 2% myeloblasts (Figure 2C). Taken together, this favored a diagnosis of CMML‐1. Chimerism testing showed 100% donor DNA in the CD3 and CD33 fractions. Cytogenetics were normal, but next‐generation sequencing showed mutations in *ASXL1* (variant allele frequency [VAF] 42%), *ETNK1* (VAF 47%), *NRAS* (VAF 46%), and *SETBP1* (VAF 48%). Testing for the *JAK2, CALR,* and *MPL* mutations was negative. There was no evidence of a *BCR‐ABL1* fusion transcript on a peripheral blood specimen. There was also no evidence of T‐ALL relapse.

The patient was treated with hydroxyurea for cytoreduction with subsequent normalization of the leukocyte count, although he continued to have a persistent monocytosis, as well as anemia requiring several transfusions. He initiated 5‐azacitidine four months later (75 mg/m^2^ on days 1‐5 every 28 days). After four cycles, he elected to discontinue 5‐azacitidine and switch back to oral hydroxyurea. A repeat bone marrow aspirate was obtained shortly thereafter and showed 15% myeloblasts, consistent with CMML‐2. Eleven months from the original diagnosis of CMML‐1, he developed worsening anemia and leukocytosis despite hydroxyurea treatment, elected to pursue hospice care, and died two months later.

## 3 DISCUSSION

The first case of DCL was reported by Fialkow et al in 1971, which described DCL arising 62 days after a matched‐related alloHCT between siblings.^4^ Herein, we describe the first reported case to our knowledge of donor cell‐derived CMML, which presented seven years after myeloablative alloHCT for T‐ALL. This case raises several interesting questions.

First, a host of recent studies have shown that clonal hematopoiesis of indeterminate potential (CHIP) is a common phenomenon that increases in prevalence with age, reaching approximately 10% in persons 70 years old.^5^ Given the increased use of matched‐related alloHCT in older adults, CHIP is likely common in older donors. Interestingly, Frick et al showed that in transplant patients whose donor had CHIP, there was a significantly elevated risk of DCL.^6^ Another study identified donor CHIP in five of six patients with unexplained cytopenias following alloHCT.^7^ Together, these reports suggest that the transference of clones harboring stereotypic CHIP mutations during alloHCT can be clinically consequential and should be further studied. Unfortunately, we were unable to test the donor's sample here for evidence of CHIP.

Second, donor cell‐derived CMML is an unprecedented phenomenon—why is unclear, but may reflect the rarity of CMML itself, which has an estimated annual incidence rate of only 0.3 per 100 000.^8^ CMML is a myelodysplastic/myeloproliferative neoplasm characterized by peripheral blood monocytosis, bone marrow dysplasia, and stereotypic mutations. While *ASXL1*, *NRAS,* and *SETBP1* mutations are frequently described in CMML, *ETNK1* mutations are present in just 2.6% of cases.^9^ CMML has a high propensity to transform to secondary AML, but there is a paucity of effective therapeutic strategies to alter the natural history of the disease. Using the Mayo Clinic prognostic model, the characteristics of this patient's CMML were poor, with an estimated median overall survival of 10 months.^10^

Due to loss of follow‐up between years three and seven post‐transplant, it is unclear specifically when this patient started showing more overt hematologic signs of CMML. Of interest, the patient's donor had polymyalgia rheumatica which, along with other inflammatory diseases, have been associated with MDS and CMML. Unfortunately, we were unable to obtain further information about the patient's brother to determine whether he had monocytosis or clinical signs of CMML in parallel.

In summary, this case report explicates the clinical history of a patient who developed donor cell‐derived CMML after alloHCT for T‐ALL, which has never been previously described. Novel therapies for CMML that improve peripheral blood counts and prevent progression to AML remain an urgent unmet need.

## CONFLICT OF INTEREST

James M. Foran: Research Support to Institution (AbbVie, Boerhinger Ingelheim, Actinium, Aprea, Aptose, H3Biosciences, Kura Oncology, Tolero, Trillium, Xencor, and Takeda/Millennium) and personal compensation (Novartis, Servier, Pfizer, and Revolution Medicine). The remaining authors declare no competing interests.

## AUTHOR CONTRIBUTIONS

Kevin C. Miller: Conception of study, literature review, data collection, and drafting of manuscript. Zaid H. Abdel Rahman: Conception of study, drafting of manuscript, and final review of manuscript. Ryan S. Robetorye: Pathologic specimen review, creation of figure, and final review of manuscript. James M. Foran: Conception of study and final review of manuscript.

## Data Availability

Not applicable; all data disclosed in manuscript.
